# Evaluation of wild herbivore faeces from South Africa as a potential source of hydrolytically active microorganisms

**DOI:** 10.1186/s40064-016-1739-y

**Published:** 2016-02-09

**Authors:** Luyanda L. Ndlela, Stefan Schmidt

**Affiliations:** Discipline of Microbiology, School of Life Sciences, University of KwaZulu-Natal, Private Bag X01, Pietermaritzburg, 3209 South Africa

**Keywords:** Fluorescein diacetate, Triphenyltetrazolium chloride, Hydrolases, Zebra, Giraffe, Impala, Nguni, Faeces

## Abstract

This study assessed faecal matter from three indigenous South African herbivores—zebra, giraffe and impala—as a potential source for hydrolytically active aerobic and facultatively anaerobic bacteria. Herbivore droppings were collected freshly in a local nature reserve in Pietermaritzburg, South Africa. Soil samples adjacent to faecal collection sites and faeces from a domestic herbivore, the Nguni cow, were included as controls. Hydrolase and dehydrogenase activity in faecal matter and soil samples were measured by the fluorescein diacetate and the triphenyltetrazolium chloride assay. Viable counts and counts for amylase, cellulase, esterase and protease producers were established using plate count agar and solid media containing cellulose, skim milk, starch and Tween 80. Zebra droppings produced the highest hydrolase and dehydrogenase activity. Faecal matter of the three indigenous herbivores generally produced higher hydrolytic activity than Nguni cow faeces and soil controls, thereby confirming that these materials are potential targets for hydrolytic enzyme mining.

## Background

Hydrolytic enzymes or hydrolases are biocatalysts that break covalent bonds by using water as co-substrate. Many hydrolytic enzymes are essential for microorganisms by enabling the utilization of organic polymers such as cellulose or starch as carbon and energy source. At the same time hydrolytic enzymes of microbial origin are applied in numerous industrial processes such as the production of food and beverage, the degradation and recycling of organic waste or the transformation of cellulosic materials into glucose for biofuel production (Bhaskar et al. 2008; Kirk et al. 2002; Gupta et al. 2002; Morrison et al. 2009). This indicates that the screening of microbes as source for such hydrolytic enzymes has economic potential. As industrial processes require enzymes able to perform optimally under specific physical and chemical parameters such as high temperatures or salinity, a potentially better option than adjusting process parameters is to search for suitable novel hydrolytic enzymes from microbial sources (Kirk et al. 2002; Cherry and Fidantsef 2003; Sanchez-Porro et al. 2003).

Faecal matter, particularly from wild herbivores such as zebra, giraffe and impala, has not been extensively studied as a source for the isolation of hydrolytic microorganisms while for domestic animals many such studies were reported (Blackburn and Hobson 1962; Varel et al. 1984; Gong 2007). A study investigating environmental sources for cellulolytic microorganisms found more than 20 different cellulolytic microbial species of which at least two were from bovine faeces (Doi 2008). Such findings highlight the potential of various faecal materials as potential sources of industrially applicable hydrolytic microbial enzymes. So far, only a few publications reported on the presence of cellulolytic hydrolytic microorganisms in zebra faeces (Sadhu et al. 2011; Laho et al. 2012). However, data are lacking on the presence of proteolytic, lipolytic or amylolytic microorganisms in zebra faeces and essentially no data are available for giraffe or impala faeces. Based on their diet comprising grasses, evergreen leaves and other shrubbery high in fibre (Pellew 1984; Keesing 1998; de Garine-Wichatitsky et al. 2004), it is highly likely that these herbivores possess active cellulolytic microbes in their faeces.

Cellulolytic microorganisms have been put under the spotlight due to the increasing need to generate renewable energy from cellulose containing organic waste (Juturu and Wu 2014). These microorganisms are involved in biofuel generation by hydrolysing cellulosic plant waste material, which is an essential part of the first hydrolysis step of the so-called anaerobic food chain. Apart from cellulases, three other major groups of hydrolases are used in industrial processes; esterases, amylases and proteases (Kirk et al. 2002; Ray 2011).

This study therefore screened fresh faecal matter of three indigenous South African herbivores, zebra (*Equus burchelli*), giraffe (*Giraffa camelopardalis*) and impala (*Aepyceros melampus*), for the presence of protease-, amylase-, esterase- and cellulase-producing aerobic and facultatively anaerobic microorganisms to verify the potential of this material as a source for such hydrolase producing microorganisms and their enzymes.

## Results

### Moisture, pH and sCOD of faeces and soil samples

The pH of fresh faecal samples including Nguni cow faeces was in a range from 7.34 to 8.32, with impala and zebra faeces having a similar, slightly alkaline pH (Table 1). In contrast, all control soil samples analysed were slightly acidic (pH range 5.81–6.34). The sCOD of zebra, giraffe and impala faeces was clearly higher (57 and 80 mg/g) than the sCOD of the matching soil samples (≤9 mg/g). This is indicative of the difference in soluble organic matter content between the collected surface soil and faeces. The cow faecal material had a much higher sCOD (339 mg/g) than the wild ungulate faeces and the highest moisture content (54 %), whilst the pH was near neutral and similar to that of giraffe faeces. Zebra faeces had the highest moisture content of the wild ungulates (49 %), while giraffe and impala faeces were almost identical with 27 and 30 % moisture content respectively. Generally, soil samples had the lowest moisture content, a more acidic pH and a much lower sCOD in comparison to the faecal samples, highlighting the apparent differences between the composition of the herbivore faeces and the soil samples.

**Table 1:** Average pH, moisture content and sCOD of freshly collected zebra, giraffe, impala and cow faeces and matching soil samples

Source	pH	Moisture content (%)	sCOD (mg/g)
Zebra faeces	8.18	49	80
Zebra soil control	5.81	4	7
Impala faeces	8.32	30	57
Impala soil control	6.34	7	9
Giraffe faeces	7.34	27	73
Giraffe soil control	5.95	4	8
Cow faeces	7.39	54	339

All data shown are the means of measurements performed on samples collected on four different occasions

### Enzymic activity of faeces and soil samples

**Fig. 1:**
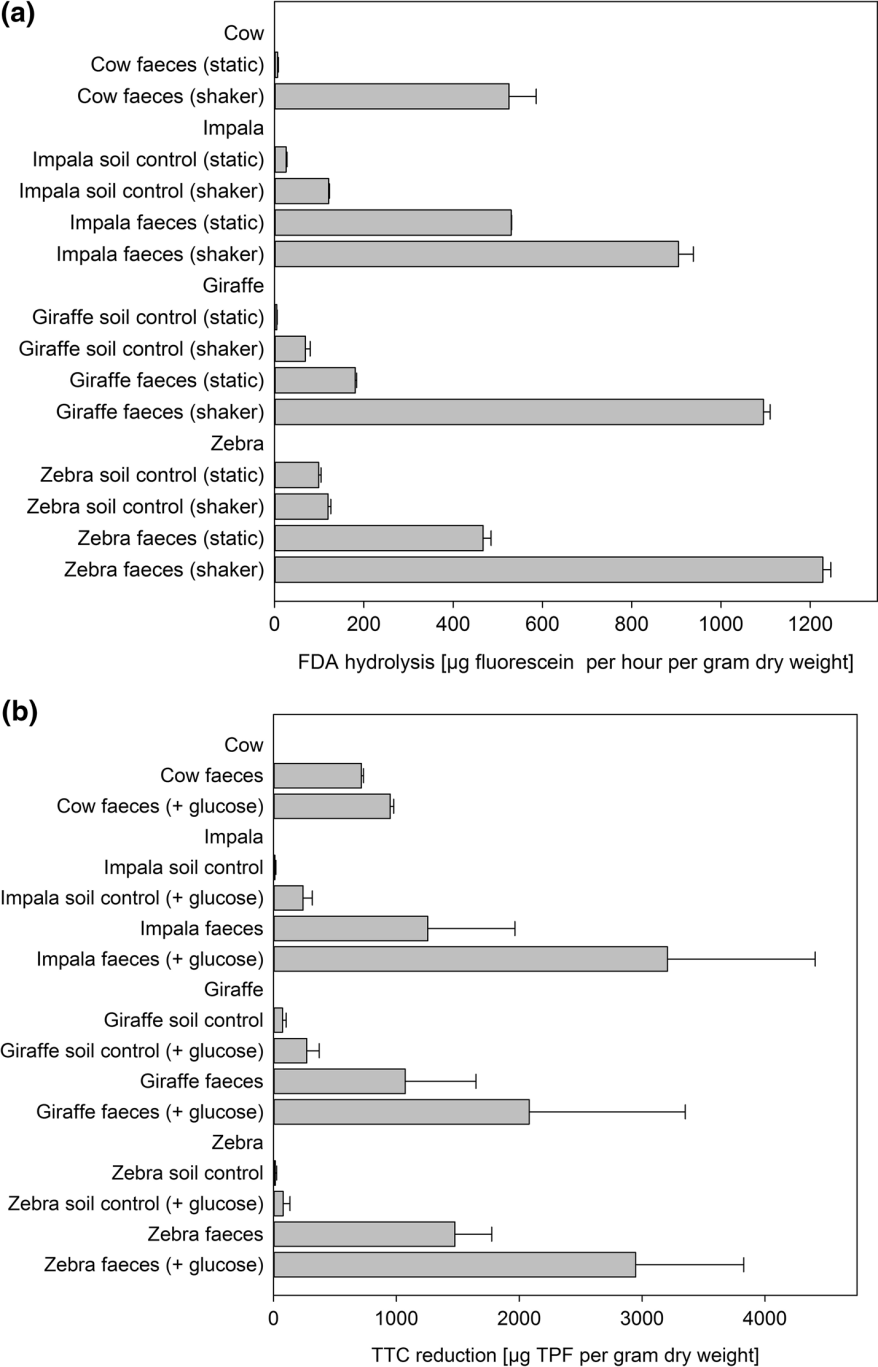
**a** Hydrolase (FDA) activities of fresh faeces and matching soil samples. **b** Dehydrogenase (TTC) activities of fresh faeces and matching soil samples. *Error bars* indicate the standard error

In the FDA assay, samples subjected to shaking during incubation showed higher hydrolytic activities than samples incubated under static conditions (Fig. 1a), which is in line with previous reports in the literature (Schnurer and Rosswall 1982). Overall, faecal samples exhibited higher hydrolytic activity than soil samples, which is expected on microbiological grounds given that the intestinal microbial community is involved in breaking down organic polymers. Under shaking conditions, zebra faeces showed the highest hydrolytic activity at 1228 µg g^−1^ h^−1^, followed by giraffe at 1095 µg g^−1^ h^−1^ and impala at 905 µg g^−1^ h^−1^. The impala soil control had the highest activity (121 µg g^−1^ h^−1^) of all control soil samples. The large differences between the hydrolytic activities of faeces and the matching soil controls highlighted the different microbial activity and abundance in these two materials. Although the Nguni cow faeces yielded a lower hydrolytic activity than the faeces of the other three ungulates analysed, its activity was higher than that of soil control samples, again indicating the expected higher abundance of hydrolytically active microorganisms in faecal matter.

Samples were also analysed for dehydrogenase activity using the TTC assay. The results (Fig. 1b) indicated that faecal samples—very much like the results obtained for the FDA assay—had a much higher enzymic activity than soil control samples. Addition of glucose to samples prior to incubation resulted in greater TPF yields via dehydrogenase activity than for samples incubated without glucose added. The highest dehydrogenase activity without glucose added was observed in zebra faeces. A comparison between the different soil controls indicated that the giraffe soil control had a somewhat higher dehydrogenase activity than the other soils. The dehydrogenase activity in Nguni cow faeces was higher than that in soils but was lower than the activity found in the faeces of the wild ungulates, which is again similar to the results obtained for the FDA assay.

### Enumeration of hydrolytic bacteria

In addition to enzymic activity, total and specific (hydrolase producing) aerobic viable counts were established (Table 2) as log_10_ cfu per gram dry weight of faeces or soil. The PC agar used is a general-purpose medium providing an estimate of the total microbial burden present whilst the different hydrolase targeting media used provide estimates for microorganisms producing cellulases, amylases, proteases and esterases. Faecal and soil samples contained all of the targeted hydrolase producers, albeit in varying proportions. The total viable plate counts (PC agar) of all faecal samples analysed were established in a similar range of log 8.13–8.53 (Table 2), with giraffe having the highest microbial count and Nguni the lowest. The total viable plate counts of surface soil samples were generally about two logs lower. Regarding the counts for different hydrolase producing bacteria, again, the faecal samples showed the highest levels and the soil samples the lowest (Table 2). However, in most cases the counts for different hydrolase producers from Nguni faeces were lower than the hydrolase producer counts obtained for wild ungulate faeces.

**Table 2:** Aerobic viable count (log_10_ cfu per g dry weight) ± standard error of fresh faecal samples and matching soil controls using plate count and hydrolase specific agar

Sample	PC agar	Starch agar	Skim milk agar	Tween 80 agar	CMC agar
Giraffe	8.53 ± 6.16	8.51 ± 6.00	8.50 ± 5.76	7.78 ± 5.08	8.40 ± 5.95
Giraffe soil	6.50 ± 3.99	6.49 ± 4.08	6.30 ± 4.25	6.20 ± 3.95	6.16 ± 3.95
Impala	8.29 ± 6.18	8.25 ± 5.76	8.24 ± 6.25	6.53 ± 3.95	8.01 ± 5.95
Impala soil	6.27 ± 3.57	6.10 ± 3.57	6.07 ± 4.50	<4	6.16 ± 4.18
Zebra	8.30 ± 5.64	8.13 ± 5.76	7.52 ± 6.32	8.16 ± 5.95	7.63 ± 4.95
Zebra soil	6.68 ± 4.18	5.92 ± 3.58	5.09 ± 3.00	<4	6.29 ± 3.50
Cow	8.13 ± 5.49	7.79 ± 5.34	6.76 ± 4.27	7.59 ± 5.36	7.47 ± 5.18

<4 = lower than detection limit of 10^4^ cfu/g dry weight

*CMC* carboxymethyl cellulose agar, *PC* plate count agar, *cfu* colony forming units

**Fig. 2:**
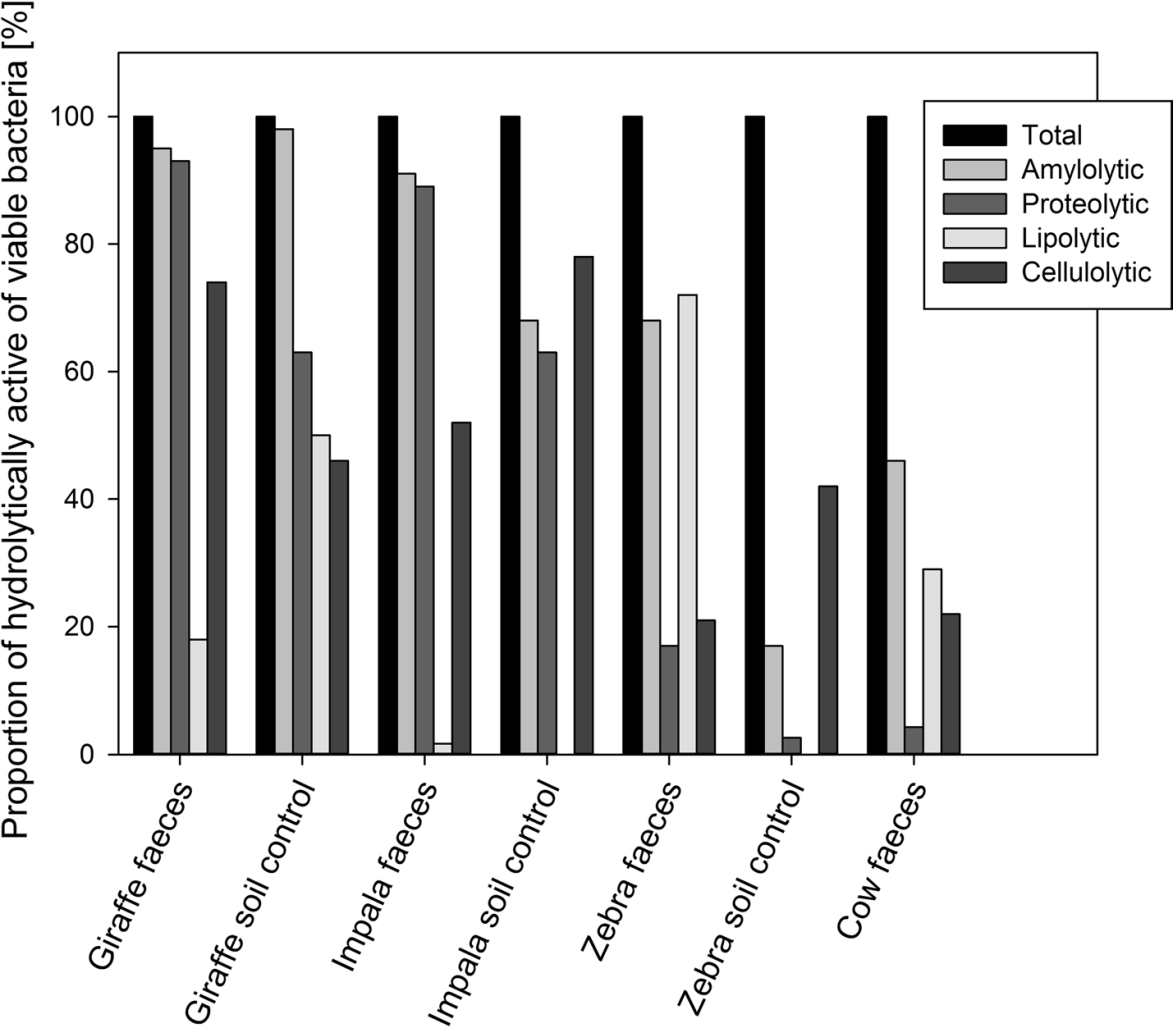
Abundance of organic polymer-hydrolysing microorganisms in fresh faeces and matching control soil samples as percentage of the aerobic plate count (100 %)

Relative abundances of specific hydrolase producing microbes were obtained by taking the colony forming units on PC agar as the total number (100 %) of viable aerobic and facultatively anaerobic microorganisms (Fig. 2). Only the giraffe soil control contained all the targeted enzyme producers while for the other two control soils the counts for esterase-producing microorganisms were below the detection limit. Faecal samples contained all the targeted hydrolase producers, with varying proportions of each hydrolase producer present in faeces of each individual animal. While giraffe and impala faeces showed similar overall proportions of hydrolase producers with the exception of lipolytic organisms which were lower in impala, both of them had higher proportions of amylase and protease-producers, with esterase-producers comprising the smallest group. Faeces of zebra and Nguni cow showed similar patterns with esterase and amylase producers as the largest and protease producers as the smallest group of the total viable count. Comparing the relative abundance of hydrolase producers and their counts to hydrolase activity determined indicated that enzymic activity was apparently linked to the overall microbial burden and not to one specific group of hydrolase producers.

## Discussion

Faecal samples analysed in this study were compared with soil collected from the same area to verify whether the surrounding surface soil differs from faecal matter regarding the microbial burden and enzymic activities. In addition, Nguni cow dung enabled a comparison of the wild herbivore faeces to that of a domestic herbivore fed on a fibrous diet.

### Properties of faeces

The neutral to slightly alkaline pH of the giraffe and Nguni cow faeces (Table 1) was within the expected pH range of ruminants, which is typically between 6 and 8 (Artan et al. 1996; Moran 2005; Marãnón et al. 2006). Zebra and impala faeces had a slightly more alkaline pH (>8), possibly indicating the presence of alkali-tolerant microorganisms therein. Soluble chemical oxygen demand (sCOD) values indicated the potential availability of substrate in the faeces enabling growth of heterotrophic microorganisms. The soil samples had a more acidic pH and much lower moisture and soluble COD content than the faecal samples at the time of sampling, matching properties reported for infertile dry soils that have a low water binding capacity (Hartemink 2006, 2007). The highest sCOD of 339 mg/g was determined for Nguni cow faeces, while the sCOD values of the other faeces ranged between 57 and 80 mg/g. These values are within the expected sCOD range of 30–6000 mg/l reported for cattle in other studies (Marãnón et al. 2006; Abubakar and Ismail 2012). The higher moisture content in faecal samples indicated that the nature of the herbivore diet was mostly water binding fibrous material due to the uptake of grasses and leaves (Ziemer et al. 2012). As a result, for all the animals analysed in this study, faecal matter had higher moisture percentage values than matching soils samples. Based on the moisture content, pH and sCOD, faecal material clearly differed from the surrounding soil.

### Analysis of enzymic activity

Microbial activity of samples was estimated through colorimetric assays that yield coloured reaction products once hydrolysis (FDA) or reduction (TTC) occurred. The fluorescein diacetate assay operates on the principle of hydrolytic release of two acetate groups via ester bond cleavage by free and membrane-bound hydrolytic enzymes, yielding the yellow-green coloured fluorescein that can be measured at 490 nm (Adam and Duncan 2001). FDA is a versatile substrate used for the detection of esterases within water bodies and individual cells (Battin 1997) and in soils (Green et al. 2006). However, other hydrolytic enzymes such as amylases can cleave its ester bonds as well (Lundgren 1981; Green et al. 2006). The FDA hydrolysis assay has also been utilised and recommended as an efficient method to estimate active cells within environmental samples (Swisher and Carroll 1980). The protocol employed in this study was optimised from Schnurer and Rosswall (1982) as suggested by Green et al. (2006) to establish the best possible assay conditions. Thus, acetone volumes used to terminate reactions were reduced to 4 % (v/v) and readings were taken within 30–60 min of termination to enable stable results (Green et al. 2006). As the FDA assay can be problematic due to abiotic cleavage caused by media components (Clarke et al. 2001; Wanandy et al. 2005), this was accounted for through use of an appropriate buffer and controls accounting for abiotic cleavage.

The FDA assay demonstrated that faecal samples from wild herbivores had higher hydrolase activity than soil controls and cow faeces, which is expected on microbiological grounds (Fig. 1a). In addition, samples incubated under shaker conditions showed higher activity than those incubated statically (Fig. 1a). Although this is contrary to the findings of Green et al. (2006) who reported that shaking decreased the amount of fluorescein released in soil samples, it confirms recommendations by Swisher and Carroll (1980) and Schnurer and Rosswall (1982) that shaking during incubation increases hydrolytic activity due to improved homogenisation. In addition, shaking improved the activity of hydrolytic microorganisms (Bozic et al. 2011) and substrate distribution (Juergensmeyer et al. 2007). The fact that a control with autoclaved faeces yielded no measurable activity in the FDA assay confirmed that its hydrolysis depended on the presence of hydrolytically active microorganisms or their enzymes in the faeces. Furthermore, the higher hydrolytic activity in faecal samples suggested that more hydrolase producing microorganisms were present in faeces than in soil as was confirmed by the plate counts (Table 2). Again, this is not unexpected as an increased degree of FDA hydrolysis relies on the presence of metabolically active microorganisms (Chrzanowski et al. 1984).

The TTC assay operates on the principle of reduction of colourless 2,3,5-triphenyltetrazolium chloride by active dehydrogenases to produce the corresponding coloured triphenylformazan (TPF). The water insoluble TPF can be extracted from samples using solvents such as methanol, butanol and ethanol and is optimally measured at 485 nm (Stevenson 1959). The assay can be used to determine cell viability (Tergendy et al. 1967) and for the analysis of microbial activity present in soils and other samples (Stevenson 1959). Like the FDA assay, the TTC assay estimates cell activity through enzyme activity. Due to its somewhat lower sensitivity, the TTC assay requires sample incubation periods of at least 24 h (Ishikawa et al. 1995). In addition, in the presence of O_2_ as a competing electron acceptor the dehydrogenase activity is potentially underestimated (Von Mersi and Schinner 1991). Therefore, samples analysed by the TTC assay in this study were incubated statically for 1 week. The TTC results are in good agreement with the findings of the FDA assay (Fig. 1). Addition of glucose confirmed the presence of glucose utilising microorganisms by leading to higher overall dehydrogenase activity due to increased microbial biomass (Fig. 1b). This is expected since gut microorganisms in ungulates ultimately break down cellulose to glucose. The high dehydrogenase and hydrolase activity of zebra faeces in the presence of a slightly elevated pH might indicate the presence of microorganisms therein able to function at higher than neutral pH, possibly indicating the presence of enzymes with potential for use in industrial applications requiring alkaline conditions (Horikoshi 1999). Comparison of sample pH values (Table 1) and TPF formation rates (Fig. 1b) indicates that increased TPF formation took place at neutral to slightly alkaline pH (i.e. pH > 7), confirming previous studies showing that the activity of electron transport systems (ETS)—including dehydrogenase activity—is enhanced within a pH range of 7.4–8 (Trevors 1984).

### Microbial counts

Viable counts in faecal samples were mostly in the range of 10^8^ cells per gram dry weight, with each faecal sample containing all targeted hydrolase producers although strictly anaerobic hydrolytically active bacteria were not quantified (Table 2). The counts for soil control samples were generally at least 1–2 logs lower than the counts in the corresponding faecal samples. In addition, clear differences in the proportions of specific hydrolase producers were evident (Fig. 2). Gong (2007) reported total and viable microscopic counts for cattle dung of about 10^11^ cfu/g (dry weight) and a matching plate count for mesophilic aerobic and facultatively anaerobic bacteria of about 10^10^ cfu/g, with proteolytic bacteria present at about 10^8^ cfu/g and both lipolytic, amylolytic and cellulolytic bacteria present at about 10^9^ cfu/g. Viable plate counts established in this study for Nguni cow faeces were lower than those reported by Gong (2007), although similar relative proportions were observed for the hydrolase producers with the exception of cellulolytic microorganisms. The difference in viable counts per g of faeces might be due to the shorter incubation time for agar plates used and the typically poorer diet of the Nguni cow (Tada et al. 2013).

With the exception of lipolytic esterase producing organisms, faecal samples from impala and giraffe showed similar proportions of amylase, cellulose and protease-producers (Fig. 2). Both impala and giraffe, ruminants as opposed to the non-ruminant zebra, had relative proportions of 91 and 95 % for amylase producers, 89 and 93 % of protease producers and fairly similar percentages of cellulase producers (52 and 74 %). Zebra in turn had the highest proportion of esterase producers (72 %) and the lowest proportion of protease and cellulase producers (17 and 21 %) amongst the three ungulates. In comparison, Nguni faeces was similar to zebra faeces with a fairly high proportion of amylase producers (46 %), a low proportion of protease producers (4.3 %) and an almost identical proportion of cellulose producers (22 %) present. Nguni faeces displayed lower hydrolytic and dehydrogenase activity compared to other ungulates, which could be due to lower proportions of hydrolase producers present and the different digestive system. These findings are consistent with previous reports in the literature suggesting that cellulolytic microorganisms when in competition with other specialists are present at low proportions (Witten and Richardson 2003). The different digestive systems present in ruminants (for example giraffe) and hindgut digesters such as zebra might result in different microbial communities.

A study of the giraffe rumen microbiome by Roggenbuck et al. (2014) showed via sequence analysis that in addition to many unknown bacteria, strictly anaerobic bacteria constitute a large proportion of the rumen community and that diet might influence the composition of this community.

The apparent differences in microbial colony counts and diversity of hydrolytically active bacteria within faeces from different herbivores (Table 2) might be due to their different feeding habits. Zebra and Nguni cattle are grazers (Odadi et al. 2011) whilst giraffe is a browser and impala is a mixed feeder (grazer and browser) (Codron et al. 2005). In Nguni cow and zebra faeces the proportion of esterase producers was higher than in faeces of giraffe and impala (Fig. 2) while the opposite applies to the proportion of cellulose producers which was lower in cow and zebra faeces than in faeces from giraffe and impala. Grazers feed more on grasses that are considered less nutritious than other forage and grazing is a lengthier process than browsing (Udén and Van Soest 1982). Zebra faeces, however, displayed a higher hydrolytic activity than Nguni cow faeces (Fig. 1a) which may be due to the difference in their digestive systems and the microbial community present. As large sized ruminants, cows have a longer digestion than non-ruminants and require more grazing time. Hydrolytic microorganisms from cattle form biofilms on forage substrates within the rumen to efficiently hydrolyse polymeric substrates (McSweeney et al. 1999) with an efficient digestion of biopolymers such as cellulose taking place in the foregut, mostly facilitated by anaerobic microorganisms. Movement of cud to the hindgut is usually required for secondary digestion. As a result, lower counts for aerobic microorganisms and hydrolase producers are expected in cow faeces (Mackie 2002). Although grass diets of cow and zebra are similar, zebra as hindgut fermenters possess an advantage by feeding on greater forage variety for shorter periods (Odadi et al. 2011). Zebra living in the local nature reserve probably had access to a more diverse diet than the Nguni cow kept at a local farm.

This study indicates that relative proportions of hydrolase producers in faeces—even though strictly anaerobic hydrolase producers were not quantified—are mostly similar for comparable feeding habits while hydrolytic activities and microbial loads can differ. This might be a result of different digestive systems and digestion times. The data for cattle and zebra indicate somewhat higher numbers of viable microorganisms in equine than in bovine faeces possibly due to shorter retention time (Odadi et al. 2011) resulting in less efficient polymer hydrolysis. As digestion occurs within the hindgut, forage is usually defecated without being properly hydrolysed resulting in more faecal shedding in zebra (Mackie 2002) than in cattle, as indicated by the results in this study.

Impala and giraffe appear to be similar regarding the relative proportions of protease, cellulose and amylase producers. As ruminants, they have a more complex digestive system than zebra and as browsers a more diverse diet. The impala in this study might have fed on a diet similar to the diet of giraffe, comprising of more browse forage than grasses, resulting in mostly similar relative hydrolase producer proportions. The smaller body size of impala means that digestion time is shorter compared to cows and faecal microbial numbers may therefore be higher (Gordon 2003).

The presence of proportionally more lipolytic esterase producers in zebra and Nguni cow than in impala and giraffe faeces might be due to the varying lipid content of plant species (Hadley and Rosen 1974) included in their diet. The presence of protease producers in faeces—although at a lower relative proportion in zebra and cow faeces—was expected, as proteases are present in the majority of heterotrophic microorganisms. Similarly, cellulose and amylase producing microorganisms were observed which is expected based on the presence of large amounts of cellulose and starch in leaves and grasses (Ben-Shahar and Coe 1992). Apart from the hydrolases targeted in this study, other hydrolytic enzymes which are of interest for industry (Kirk et al. 2002) such as xylanases and hemicellulases are typically detected in faecal samples of ungulates (Fon and Nsahlai 2012).

Microbial interactions in the intestinal systems of these herbivores are similar to anaerobic bioreactors utilizing cellulosic material as substrate, with similar hydrolytically active bacteria documented as members of the anaerobic food chain in artificial (bioreactors) and natural systems (digestive tract of herbivores) (Krakat et al. 2011; Morrison et al. 2009).

## Conclusions

The detection of large numbers of proteolytic, cellulolytic, amylolytic and lipolytic bacteria in faecal matter from zebra, giraffe and impala indicates that this material is a useful source for the isolation of hydrolase producing microorganisms. Zebra, giraffe and impala faeces appeared more promising than cattle faeces and soil controls due to generally higher numbers of microbial hydrolysers and higher overall hydrolytic activity. This study used a culture-based approach to screen for the presence of specific hydrolase producers present in herbivore faeces. A metagenomics approach targeting genes encoding for hydrolytic enzymes in these samples could be a potential experimental technique to evaluate such faeces as source for additional hydrolases.

## Methods

### Sample collection

Fresh faecal samples from zebra, giraffe and impala were collected on four different occasions (January 2011–January 2012) at the Bisley Valley Nature Reserve in Pietermaritzburg (S29°39′44″, E30°23′25″). Soil samples near faecal collection points and fresh faeces from pasture-fed Nguni cows (Ukulinga Research Farm, UKZN, Pietermaritzburg) were used for comparison. All soil and faecal samples were collected in sterile plastic bags, transported on ice and then stored at 4 °C. Samples were analysed in the laboratory on the same day within 6 h after collection.

### Moisture content

About 1 g of sample material was dried in an oven at 105 °C for 48 h. Samples were then cooled to room temperature, reweighed and the percentage moisture content was established.

### Soluble chemical oxygen demand

The soluble organic fraction in fresh faeces and soil samples was quantified as soluble chemical oxygen demand (sCOD). 100 fold dilutions of 1 g of fresh sample material in distilled water were homogenised (shaking at 150 rpm for 20 min), centrifuged (5 min at 10,000×*g*) and then used to determine the sCOD as reported previously (Gemmell and Schmidt 2012). Values were established as mg of sCOD per gram dry weight.

### Measurement of pH

The pH of samples was measured according to ISO 10390 (2005) by adding 50 ml of 0.1 M calcium chloride solution to 10 g of air-dried (48 h in the dark) homogenised sample material. Measurements were done using a calibrated pH electrode (Crison, MicropH 2001, USA) after 5.5 h incubation at 25 °C.

### Hydrolase and dehydrogenase activity

Overall hydrolytic activity in samples was quantified using a modified fluorescein diacetate (FDA) assay essentially following the procedure reported by Green et al. (2006), while the dehydrogenase activity in samples was determined using the triphenyltetrazolium chloride (TTC) assay (Stevenson 1959). The FDA assay was carried out in duplicate Erlenmeyer flasks containing 50 ml 60 mM sodium phosphate buffer (pH 7.6), 0.5 ml FDA (4.8 mM in acetone) and 1 g of fresh sample material followed by incubation for 1.5 h in the dark at 30 °C, either statically in a thermo-controlled incubator or at 150 rpm in a thermo-controlled shaker. Reactions were terminated by adding 2 ml acetone, followed by centrifugation (20,000×*g*, 5 min), measuring the absorbance at 490 nm (Shimadzu 1240) and extrapolating fluorescein concentrations using calibration curves established using authentic fluorescein. Hydrolase activities were established as µg of fluorescein formed per gram dry weight per hour of incubation. Appropriate controls (flasks with sample material and no FDA and flasks with only phosphate buffer and FDA) were always included and accounted for. For the TTC assay 7.5 ml deionised water, 3 ml 3 % aqueous TTC and 6 g of fresh sample were mixed and incubated for 1 week in the dark at ambient temperature. Additional incubations were done with 0.1 g glucose added as substrate to verify the presence of glucose utilizing microorganisms. Triphenylformazan (TPF) was extracted from samples using methanol and quantified with absorbance measured at 485 nm and by using standard curves established using authentic TPF. The dehydrogenase activities were reported as µg of TPF formed per gram dry weight.

### Bacterial enumeration

Viable counts of heterotrophic bacteria were determined via spread plating onto plate count (PC) agar (Merck). Decimal dilutions of environmental samples (ranging from 10^−1^ to 10^−8^) were established by initially adding 10 g of sample material to 90 ml peptone water (8.5 g NaCl and 1 g peptone per litre, pH 7.0) followed by homogenisation at 150 rpm for 15 min and subsequent decimal dilutions up to 10^−8^. Samples (100 µl) from each decimal dilution were then spread-plated in triplicate onto PC agar; Tween 80 agar (5 g peptone, 3 g meat extract, 10 ml Tween 80, 100 mg CaCl_2_ × 2H_2_O and 15 g agar per litre, pH 7.2); skim milk agar (10 g skim milk powder, 3 g meat extract, 5 g NaCl, 2 g Na_2_HPO_4_, 15 g agar and 0.05 g bromothymol blue per litre, pH 7.2); carboxymethylcellulose agar (CMC agar) (2 g NaNO_3_, 1 g K_2_HPO_4_, 0.6 g MgSO_4_, 0.6 g KCl, 2 g carboxymethylcellulose sodium salt, 0.2 g peptone and 17 g agar per litre, pH 7.2) and starch agar (3 g beef extract, 10 g soluble starch and 12 g agar per litre, pH 7.2). Colony counts were established after incubation for 48 h at a temperature of 30 °C as suggested by Gong (2007). CMC and starch agar plates were flooded with Gram’s iodine solution to detect cellulase and amylase positive colonies displaying a clear halo; protease positive colonies displayed blue colour due to casein hydrolysis on skim milk agar; esterase positive colonies on Tween 80 agar produced calcium oleate precipitates.
